# The Battle beyond the Battlefield: War’s Influence on Antibiotic Resistance

**DOI:** 10.3390/idr16050077

**Published:** 2024-10-09

**Authors:** Guido Granata, Stefania Cicalini, Nicola Petrosillo

**Affiliations:** 1 Systemic and Immune Depression-Associated Infection Unit, National Institute for Infectious Diseases “L. Spallanzani”, IRCCS, 00149 Roma, Italy; 2 Infection Prevention & Control/Infectious Disease Service, Fondazione Policlinico Universitario Campus Bio-Medico, 00127 Rome, Italy

In July 2024, poliovirus was identified in Gaza, prompting the World Health Organization (WHO) to issue a warning regarding the potential for polio to spread in the region [1]. The Gaza Strip has been free of polio for the past 25 years. The re-emergence of polio now represents a significant threat to the children in the Gaza Strip and neighboring countries. Consequently, the WHO and the United Nations Children’s Fund (UNICEF) have jointly called for a seven-day humanitarian ceasefire in the Gaza Strip to facilitate the administration of polio vaccines [2]. Furthermore, the WHO has stated that a permanent ceasefire is the only means of ensuring public health security in the Gaza Strip and the wider region.

In addition to the ongoing threat of polio, there is concern over the potential rise of other infectious diseases, which could result from a combination of factors, including clean water shortages, overcrowding, and a lack of access to sanitation and hygiene. Cholera is an acute diarrheal illness caused by intestinal *Vibrio cholerae* infection [3]. Given its fecal–oral transmission, cholera often accompanies humanitarian crises and can be spread by contaminated water supplies. The development of cholera infection can lead to severe dehydration and, if untreated, shock, coma, and death within hours [3]. Cholera outbreaks can rapidly evolve and require efficient surveillance and intervention. The prevention and control of cholera outbreaks is multifaceted, needing a combination of water sanitation, hygiene, and treatment. A cholera outbreak is possible in war areas, such as the Gaza Strip, considering the high summer temperatures and the difficulties in delivering bottled or purified water in the region.

Furthermore, the prevalence of bacterial infections caused by multidrug-resistant (MDR) bacteria in the context of armed conflicts represents a growing concern [4,5]. Over the past half-century, there has been a correlation between geopolitical conflicts and the emergence of antimicrobial resistance [5].

Studies performed during the conflicts in Iraq and Afghanistan reported a significant increase in the incidence of carbapenem-resistant *A. baumannii* isolates compared to the period before the conflict [6,7]. A study reported the presence of carbapenem-resistant Gram-negative isolates and MRSA in 37.3% and 16.4% of the 67 soldiers transferred to Germany after the Libyan war, respectively [8]. The production of metallo-β-lactamase, OXA, and other β-lactamases was reported among isolates from the Libyan war [8,9]. One of the earliest reports of New Delhi metallo-β-lactamase-producing enterobacteria came from the conflicts in Libya [8].

Reports indicate that enterobacteria have notably increased during the ongoing conflict in Ukraine, producing metallo-β-lactamase and OXA-48 [10]. A recent study evaluated 154 isolates from patients injured during the war in Ukraine [10]. Among the isolates, 89 (58%) were resistant to carbapenems. Furthermore, extended antimicrobial susceptibility testing indicated that 49% of the tested strains were cefiderocol resistant. The resistance rates for other novel antibiotics were also higher than 80% for ceftazidime-avibactam, ceftolozane-tazobactam, imipenem-relebactam and meropenem-vaborbactam [10]. Alarmingly, 6% of the isolates, all of which were *K. pneumoniae*, were pandrug-resistant. Genetic screening identified a prevalence of New-Delhi metallo-β-lactamase and OXA-lactamases [10].

Research from previous conflicts has consistently shown the isolation of strains resistant to the most recent antibiotics available, predicting the worldwide proliferation of these MDR pathogens. In this context, the elevated prevalence of New-Delhi β-lactamase serves as a concerning indicator of the potential global spread of pandrug-resistant Gram-negative pathogens, exacerbated by the ongoing wars.

Recent studies from the Dutch and German surveillance databases corroborated the concern about the global spread of pandrug-resistant bacteria [11,12]. A large retrospective microbiological study reported that the high rates of MDR and pandrug-resistant bacteria among war refugees determined a significant rise in the incidence of New Delhi metallo-β-lactamase-producing *K. pneumoniae* in Germany, compared to the baseline incidence (*p* < 0.001) [12].

Several hypotheses can be drawn regarding the causes of antimicrobial resistance development during armed conflicts. First, the damage to laboratory facilities, often insufficient in many conflict zones, can hinder microbial testing, including tests for antimicrobial susceptibility and the appropriate use of antibiotics [13,14].

Second, the high incidence of traumatic bone and soft-tissue injuries in individuals exposed to the conflict underscores the necessity for damage-control surgery. However, these procedures are often carried out in facilities that do not have standard infection control measures, raising the risk of wound contamination [15,16]. Injuries sustained by soldiers in combat frequently lead to devitalized and contaminated tissue, resulting in a higher risk of infection and complications, as well as promoting the emergence of antimicrobial resistance. It is paradoxical that to manage these challenges, healthcare providers often use broad-spectrum antibiotics, which can also contribute to developing antibiotic resistance by selecting resistant bacteria [17,18].

Moreover, the high incidence of MDR *A. baumannii* during recent armed conflicts may be linked to integrons containing antibiotic-resistance genes. Possessing integrons with multiple resistance determinants may confer a strong selective advantage in an environment where antibiotics are frequently used [7].

Additionally, inadequate disinfection practices and environmental transmission are likely key contributors to the spread of *Acinetobacter* in military hospitals [7]. If soil inoculation at the time of injury had constituted the primary source of infection, it would be expected that most MDR isolates would be derived from wound samples. However, many isolates from non-wound sources were reported in the available studies, which suggests that introducing the organism from environmental contamination at the time of injury is an unlikely cause of MDR infection [19,20,21,22,23]. The available surveillance studies that employed molecular and genetic characterization provide evidence regarding the nosocomial spread of antibiotic resistance during wartime and suggest that transmission within military healthcare facilities plays a key role [19,20,21,22,23].

It is crucial to note that adherence to practice guidelines and improved antibiotic stewardship can reduce the risk of MDR infections among military personnel [24]. A single-center study indicated that active surveillance and infection control measures can minimize the nosocomial transmission of MDR bacteria in military hospitals [25].

In order to address the challenge of war’s influence on antibiotic resistance, it is crucial to implement educational initiatives targeting healthcare providers in military hospitals. These initiatives should encompass antibiotic stewardship, including promoting proper antibiotic use and awareness of the risks associated with antibiotic resistance. The choice of first-line antibiotics should be based on the culture obtained on admission to the hospital [26]. Furthermore, it is imperative to implement rigorous nosocomial infection control measures.

During armed conflicts, emergency medical care is frequently provided by various organizations, including the redeployed local workforce, the United Nations, the WHO, allied nation militaries, and non-governmental organizations. Active collaboration is needed to optimize the potential for surveillance and infection control. An additional level of international strategy, specifically aimed at supporting countries in conflict, would be beneficial. This should also include infection control training where necessary.

These steps can help to mitigate the risk of antibiotic resistance during wars and safeguard the efficacy of antibiotics for future generations.
